# Knowledge and perceptions of genetic testing for patients with breast cancer in Nigeria: a survey of healthcare providers

**DOI:** 10.1186/s13053-025-00315-w

**Published:** 2025-05-19

**Authors:** Funmilola Olanike Wuraola, Anna Dare, Jenine Ramruthan, Emma Reel, Anna T. Santiago, Folorunso Sharif, Agodirin Olayide, Nneka Sunday-Nweke, Olusegun Alatise, Tulin D. Cil

**Affiliations:** 1 Department of Surgery, Obafemi Awolowo University, Ile-Ife, Nigeria; 2 Department of Surgery and Dalla Lana School of Public Health, University of Toronto, Toronto, Canada; 3 Department of General Surgery, University Health Network, Toronto, Canada; 4 Department of Biostatistics, University Health Network, Toronto, Canada; 5 Department of Radiology, Obafemi Awolowo University Teaching Hospitals Complex, Ile-Ife, Nigeria; 6 Department of Surgery, University of Ilorin, Ilorin, Nigeria; 7 Department of Surgery, Alex Ekwueme Federal University Teaching Hospital, Abakaliki, Nigeria; 8 Department of Surgery, University of Toronto, Toronto, Canada; 9 Princess Margaret Cancer Centre, 700 University Avenue, Toronto, Canada

**Keywords:** Hereditary genetic testing, Breast cancer, Healthcare providers, Low-and middle-income countries

## Abstract

**Background:**

The role of genetics in breast cancer management is becoming increasingly essential in sub-Saharan Africa (SSA). Harmonized Guidelines by the National Comprehensive Cancer Network (NCCN) for SSA outline the subset of patients requiring genetic testing for hereditary breast cancer as part of their treatment plan. However, in low-and middle-income countries (LMICs) like Nigeria, access to genetic counselling and testing remains limited. Additionally, the knowledge and acceptability of these available services from the healthcare provider (HCP) perspective are largely unknown. This study aimed to assess the knowledge and perceptions of hereditary breast cancer testing among HCPs in Nigeria.

**Methods:**

In June 2022, we conducted a survey among 549 Nigerian HCPs. The 35-item survey was administered using Google Forms and distributed via WhatsApp. The survey collected demographic data and included three sections on genetic testing in breast cancer patients, focusing on knowledge, perceptions, and training.

**Results:**

The results were analyzed using R Version 4.4.1 (R Core Team). Altogether 121 HCPs responded (22% response rate): 54 (44.6%) general surgeons, 4 (3.3%) breast surgical oncologists, 29 (24.0%) clinical and radiation oncologists, 31(25.6%) oncology nurses, and 3 (2.5%) breast radiologists. The survey results indicate that Nigerian HCPs were knowledgeable about hereditary breast cancer genetics, but the implementation of counselling and testing was low. Only 32.2% of respondents had requested genetic testing for their patients, and all testing was done through private laboratories. Only 9.9% had received formal clinical genetics training, and 13.2% reported having a genetic counsellor in their hospital. There was considerable interest in future genetics training programs using in person and online teaching modalities.

**Conclusion:**

This survey highlights the need for specialized breast cancer genetic training tailored for Nigerian HCPs, which is essential in achieving breast cancer treatment parity. Addressing the substantial challenges in expanding genetic testing capacity in Nigeria is warranted for future progress.

**Supplementary Information:**

The online version contains supplementary material available at 10.1186/s13053-025-00315-w.

## Background

Nigeria is the most populous country in Africa and breast cancer is the most common female malignancy, with an incidence rate of approximately 54.3/100,000 [1] which has steadily increased. Among Nigerian women, breast cancer is diagnosed at a younger age (15–39 years) and later stage (> 80% with stage III/IV) [2, 3]. A higher proportion of patients have tumours that are triple negative (~ 40%) and both overall and stage-specific survival is worse in Nigeria than in high-income countries [3, 4].

Genetic counselling and testing are essential in the era of personalized breast cancer management. While these services are the standard of care in most high-income countries, their integration remains inadequate in low- and middle-income countries (LMICs) like Nigeria, where resources aimed at early detection and prevention of breast cancer are limited. Since breast cancer constitutes up to 22.7% of all new cancer diagnoses in Nigeria [5], its increasing incidence and high mortality rates underscore the urgent need for improved diagnostic and treatment approaches in this population.

The most common heritable mutations associated with increased breast cancer risk are those in the *BRCA1* and *BRCA2* genes [6]. The prevalence of these pathogenic variants varies by population and remains poorly characterized outside of high-income populations [7, 8]. In developed countries, genetic testing and counselling are widely and commercially available to evaluate the lifetime risk of hereditary breast and ovarian cancer in high-risk individuals and families, informing subsequent disease management [9]. In recent years, gene panel testing has become the standard of care and is recommended by most guidelines for patients with breast cancer based on select criteria [6, 10]. The 2024 American Society of Clinical Oncology (ASCO) guidelines recommend that *BRCA1/2* testing be offered to every patient newly diagnosed with breast cancer under 65 years, and selected patients over 65 moving towards universal testing [10]. The shift towards broader testing aligns with the clinical benefit of early therapeutic management of these pathogenic variants and the effectiveness of risk reduction strategies. The NCCN recommends genetic testing based on the following criteria: age at diagnosis below 50 years of age, triple negative breast cancer, personal history of breast cancer, family history of breast cancer, treatment indication, or multiple cancer diagnoses, male breast cancers, and those with Ashkenazi Jewish ancestry [6]. While these personal and family history-based guidelines have been valuable in sub-Saharan Africa (SSA), the ASCO guidelines now emphasize the importance of broader testing to capture a wider range of variants and improve clinical outcomes for all patients with breast cancer. This is especially important in SSA countries such as Nigeria where more than half of newly diagnosed patients are under 50 years of age and have a high proportion of triple-negative breast cancer [3, 11]. Identifying a pathogenic variant can significantly influence the management options available to the carrier, as well as for asymptomatic relatives who may also be at risk.

In Nigeria, however, the understanding of hereditary breast cancer gene variant prevalence, penetrance, and relative risk of associated cancers is limited. Globally, it is estimated that 5–10% of all breast cancers are associated with an inherited mutation in a high risk cancer predisposition gene [12]. However, little is known about the incidence and prevalence of these pathogenic variants in the Nigerian population. A single institution case control study in Ibadan, Nigeria, reported that one of eight participants with invasive breast cancer had a pathogenic variant in a high-risk breast cancer gene [13]. This study reported the *BRCA1* and *BRCA2* carrier prevalence to be as high as 7.0% and 4.1%, respectively. In comparison, population-based studies among black patients with invasive breast cancer from North America have reported a combined *BRCA* prevalence ranging from 4% [14] to 12.4% [15]. Combined with the extremely high risk of breast cancer associated with these pathogenic variants (40–90%) [16], the high prevalence of *BRCA1/2* in Nigeria underscores the critical role of genetic testing for early cancer detection and treatment.

Several factors might account for the low rate of genetic testing in Nigeria, including lack of knowledge and awareness (patient and provider), the absence of genetic counselling services, and the lack of specialized testing facilities. Previous studies conducted in Ibadan have explored perspectives on genetic testing and counselling for breast cancer among professional women (female bankers and university lecturers) without a personal history of breast cancer as well as patient populations. The data from these patient-facing studies highlight a strong interest in genetic testing services and a high intention to use these services, despite a general lack of knowledge about breast cancer genetics [17–20]. Collectively, these findings underscore the importance of integrating genetic services for effective cancer risk management into LMICs such as Nigeria. However, before introducing or expanding breast cancer genetic counselling and testing services in Nigeria, it is crucial to understand the existing challenges related to these procedures from the viewpoint of healthcare providers (HCPs). Therefore, this study aimed to assess the knowledge, perceptions, and training related to genetic testing for hereditary breast cancer among HCPs in Nigeria.

## Methods

Healthcare providers specializing in breast cancer care across all geopolitical zones in Nigeria were invited to participate in this survey using a convenience sampling approach. A 35-item (unvalidated) questionnaire was developed based on clinical observation and a review of existing literature. The questionnaire included 9 demographic questions, and three main sections related to genetic testing in patients with breast cancer: knowledge (4 questions), perceptions and barriers (15 questions), and genetics training (6 questions). The format of questions included multiple-choice items, five-point Likert scales, and open-ended responses (Supplementary Material 1).

The survey was created on Google Forms and distributed to HCPs using WhatsApp™, a widely used social media platform among HCPs in Nigeria. Consent was obtained through the administrators of several professional WhatsApp groups representing breast cancer care providers nationally, including the Nursing Oncology, General Surgery, and Clinical Oncology groups. The survey link was shared within these groups, ensuring participant privacy, and allowing for voluntary participation at the respondents’ convenience. While WhatsApp is a popular and accessible platform in Nigeria, we acknowledge that some providers may have been excluded if they were not members of these groups, were inactive on the platform, or had muted notifications, which could limit reach. Data collection was ongoing for four weeks, during which weekly reminders were sent to encourage participation. Ethical approval was obtained from Obafemi Awolowo University Teaching Hospitals Complex, Ile-Ife, Nigeria.

### Data analysis

Providers’ sociodemographic data (i.e., gender, age, types of practice settings, HCP group, geopolitical zone, number of patients with breast cancer seen per month) and responses to questionnaires pertaining to HCP knowledge of indications for hereditary genetic testing of breast cancer, perceptions of hereditary breast cancer diagnosis, and barriers to conducting genetic testing were summarized using descriptive statistics including frequency and percentages for categorical responses, or mean, standard deviation (sd), median, and range for numerical ratings. Associations between sociodemographic characteristics and responses to questionnaires were assessed using the Fisher’s exact or Chi-Squared tests. Statistical analysis was performed using R Version 4.4.1 (R Core Team).

## Results

### Sociodemographic characteristics

Five hundred forty-nine HCPs were invited to participate in the survey between June 1st and June 30th, 2022, of which 121 HCPs (22%) completed all questions and were included in the analysis. The breakdown of responses by discipline was as follows: 54 (44.6%) general surgeons, 4 (3.3%) breast surgical oncologists, 29 (24.0%) clinical and radiation oncologists, 31 (25.6%) oncology nurses, and 3 (2.5%) breast radiologists. Of the physicians, 58.9% were consultants, while 41.1% were senior residents. Most respondents (*n* = 96, 79.3%) were employed at teaching hospitals across all geopolitical zones of the country. The South West region had the highest representation with 47 (38.8%) respondents, followed by the North Central region with 30 (24.8%). The North East region had the lowest number of respondents, with only 6 (5.0%). The majority of participants (*n* = 79, 65.3%) were between 31 and 44 years of age. The gender distribution was 43 (35.5%) female, 77 (63.6%) male, and 1 (0.8%) who preferred not to disclose their gender (Table 1).

**Table 1:** Sociodemographic distribution of the study respondents

	*n* = 121
Gender	
Male	77 (63.6)
Female	43 (35.5)
Prefer not to say	1 (0.8)
Age	
* ≤*30years	4 (3.3)
31-44years	79 (65.3)
45-64years	37 (30.6)
* ≥*64years	1 (0.8)
Types of Practice Settings	
Private	8 (6.6)
Public/Teaching	96 (79.3)
Public/Non-Teaching Hospital	17 (14.0)
Healthcare Provider Group	
Breast Surgical Oncologist	4 (3.3)
General Surgeon	54 (44.6)
Clinical and Radiation Oncologists	29 (24.0)
Breast Radiologist	3 (2.5)
Nurse Oncologist	31 (25.6)
Geopolitical Zone	
North Central	30 (24.8)
North East	6 (5.0)
North West	21 (17.4)
South South	8 (6.6)
South West	47 (38.8)
South East	9 (7.4)
Number of patients with breast cancer seen per month	
1–10 patients	52 (43.0)
11–20 patients	39 (32.2)
* ≥* 21patients	30 (24.8)

### Description of practice

The survey also examined the practice settings of the respondents, focusing on the type of hospital where they work, the volume of patients with breast cancer seen per month, and the available referral pathways for genetic counselling. When asked about the volume of patients with breast cancer within their practice, a significant portion of participants (*n* = 52, 43.0%) reported that they attend to between 1 and 10 patients with breast cancer monthly, while 30 HCPs (24.8%) see more than 21 patients per month (Table 1). Regarding the availability of genetic counselling services, over half of the participants (*n* = 70, 57.8%) indicated that they did not have provision for genetic counselling, and majority (*n* = 85, 70.2%) reported having no pathway for referral.

### Knowledge of hereditary breast cancer

In this section, the survey assessed participants’ knowledge by asking them to identify the criteria for hereditary breast cancer genetic testing as outlined by the National Comprehensive Cancer Network (NCCN) recommendations. Most respondents accurately identified the following: 70.2% (*n* = 85) chose those with a family history of cancer, 77.7% (*n* = 94) identified those with multiple malignancies, 76.9% (*n* = 93) indicated those diagnosed under 50 years of age, and 66.1% (*n* = 80) selected those with triple-negative breast cancer (Table 2). Overall, 58.7% of respondents correctly identified all NCCN-recommended criteria, achieving a perfect score (100%). Further analysis showed no significant associations between demographic characteristics and knowledge of hereditary breast cancer genetic testing (Supplementary Table 1).

**Table 2:** HCP knowledge of indications for hereditary genetic testing of breast cancer

	*n* = 121(% correct)
Family history of breast cancer	85 (70.2)
Multiple malignancies	94 (77.7)
Younger than 50 years of age	93 (76.9)
Triple negative breast cancer	80 (66.1)
All of the above	71 (58.7)
None of the above	0 (0)

### Perceptions of hereditary breast cancer

When asked if *BRCA* testing will influence breast cancer care in Nigeria, nearly all respondents (*n* = 113, 93.4%) affirmed that genetic testing has immense prognostic and predictive benefits. They noted that hereditary testing could assist in early detection of breast cancer, identification of at-risk individuals, and implementation of early preventive measures such as risk reduction mastectomy and chemoprevention. Additionally, participants acknowledged the importance of genetic counselling for educating patients and their family members about preventive measures and follow-up screening for improved outcomes. Many respondents (62.5%) found that a hereditary breast cancer diagnosis is useful to them in patient management (Table 3). The average score of 1.8 (SD 1.2) suggests that most respondents found information regarding a hereditary breast cancer diagnosis to be beneficial. Notably, the perceived usefulness was consistent across demographic variables, with no significant associations identified (Supplementary Table 2).

**Table 3:** Perceptions of a hereditary breast cancer diagnosis

	*n* = 121(%)
1 - Useful	75 (62.5)
2 - Somewhat useful	16 (13.3)
3 - Neutral	13 (10.8)
4 - Somewhat not useful	11 (9.2)
5 - Not useful	5 (4.2)
Perception numeric	
Mean (sd)	1.8 (1.2)
Median (Min, Max)	1 (1, 5)
Not reported	1

### Barriers to inclusion of genetic testing in clinical practice

Less than half of all respondents, (*n* = 53, 43.8%) routinely discussed genetic testing with their patients. The remaining half indicated that the unavailability of testing facilities deters them from discussing genetic testing. In a multiple-response question, the barriers to conducting genetic testing reported by HCPs were a lack of funding and testing centres as noted by 56% (*n* = 68) and 47% (*n* = 57) of respondents respectively. Conversely, awareness of genetic testing and patient interest was not widely viewed as an obstacle. Interestingly only 2% (*n* = 3) noted the lack of a genetic counsellor as a barrier (Table 4). Results were consistent across demographic groups, with no differences observed (data not shown).

**Table 4:** Barriers to conducting genetic testing

	*n* = 121 (%)
Not aware of need for genetic testing	8 (7)
Lack of funding	68 (56)
Lack of testing centres	57 (47)
Patient not interested	18 (15)
No reason	3 (2)
No genetic counsellor	3 (2)

Only 16 (13.2%) respondents had access to genetic counsellors, with the highest availability noted in the South West, followed by the North West zones (Table 5). There was also variability in the consistency with which family history information was collected. Using a 5-point Likert Scale ranging from never, occasionally, sometimes, often and always, only 27 (22.3%) consistently collected a complete family history, including three-generation disorders, and the age at diagnosis and death of each affected family member, while 14 (11.6%) have never collected a complete family history. Regarding referrals for genetic testing, about one-third (*n* = 39) of respondents have experience in requesting genetic testing in their practice. All testing was performed in private laboratories, with SYNLAB being the preferred local option [21]; other patients were sent to specialized facilities in major cities such as Lagos and Abuja, or abroad.

**Table 5:** Reported availability of genetic counsellors across geopolitical zones in Nigeria

Geopolitical Zone	Availability of Genetic Counsellors
North Central	2
North East	0
North West	5
South South	0
South East	0
South West	9

### Training

Out of 121 respondents, only 12 (9.9%) received formal genetics training. Among these only, 1(0.8%) indicated having online training, 3 (2.5%) had on-site training by a group of professionals in a university hospital in Nigeria, and 5 (4.1%) underwent postgraduate training. Almost all participants who had no formal training expressed willingness to undergo training or nominate a colleague from their institution to do so. Preferences for training modes were similar: 33.9% preferred hybrid or in-person formats, and 32.2% preferred online. Additionally, 87 (71.9%) believed that a three-month training period would be sufficient.

## Discussion

Our findings provide insights into the current state of genetic services for breast cancer across Nigeria and highlights significant gaps in the access and utilization of genetic testing and counselling. The survey engaged 121 HCPs across different cadres who regularly manage patients with breast cancer. This approach ensured a well-rounded representation of perspectives. The findings indicate that while HCPs demonstrate a reasonable level of knowledge regarding the NCCN-recommended eligibility criteria for hereditary breast cancer genetic testing there was a reasonable proportion that did not correctly identify *all* of the necessary patient criteria based on the outlined risk factors. Notably, no significant associations were observed between level of knowledge and demographic characteristics. These findings point towards the need for targeted educational interventions, particularly addressing the under-recognized indications for genetic testing (such as triple-negative breast cancer) as highlighted in our study.

Next, we investigated HCP perceptions of hereditary breast cancer. The majority of respondents indicated that diagnosing hereditary breast cancer would be useful in their clinical practice. Notably, the perceived usefulness was consistent across various demographic groups, with no significant associations identified between respondents’ backgrounds. This indicates Nigerian HCPs widely recognize the utility of hereditary breast cancer management, regardless of their role, years of experience, practice setting, or region. Building on this, almost all respondents recognized the importance of *BRCA* testing and risk management for improving the prognosis of affected individuals and reducing breast cancer associated morbidity and mortality. Participants also noted the benefits for family members sharing the same hereditary predisposition, including increased surveillance which can lead to earlier risk reduction surgery and improved survival outcomes. Surgical options such as bilateral mastectomy and risk-reducing salpingo-oophorectomy (RRSO) are the most effective method of reducing cancer risk among *BRCA* carriers [22]. However improved patient education on the benefits and implications of early risk reduction surgeries is essential in Nigeria, given the low levels of patient acceptance and understanding [23]. In contrast, patients in high resource settings are increasingly opting for risk reduction mastectomies, guided by their genetic test results [24].

HCPs identified a substantive shortage of genetic counselling services in our study. Many reported a complete lack of genetic counselling services and resources within their practice settings, and most noted the absence of referral pathways for genetic assessments. Less than half of all respondents engage in routine discussions about genetic testing with their patients. The highest availability of genetic counsellors was noted in the South West region, which is unsurprising, based on previous studies by the Ibadan group [18]. Despite this regional availability, the national shortage of trained genetic counsellors remains a critical barrier to managing hereditary breast cancer in Nigeria. However, the most frequently reported barriers to implementing genetic testing from the viewpoint of HCPs were financial constraints and the lack of testing centres. This is common among LMICs, as the high cost of genetic testing, combined with the absence of insurance coverage or governmental support, places the financial burden directly on patients and impedes uptake. Even in high-income countries, testing relies on subsidies or financial aid programs [25]. In contrast, HCPs did not view awareness of genetic testing, patient disinterest, and lack of genetic counsellors as significant barriers. The low prioritization of genetic counsellors may imply several underlying dynamics. It may reflect a limited understanding of the role that structured genetic counselling can play in patient care, given the lack of exposure to genetic counsellors in the Nigerian setting. Alternatively, it may suggest that practitioners feel sufficiently prepared to integrate genetic counselling into their routine practice, effectively mainstreaming these services without the need for a genetic counsellor. This type of education and integration approach may be pragmatic in regions where genetic counselling resources and personnel are limited. Addressing these constraints and enhancing provider training could be pivotal for expanding the reach and efficacy of genetic testing services in this setting.

According to our survey only a minority of HCPs had experience in consenting or routinely ordering genetic tests for their patients. Our findings also noted that most HCPs often rely on external private laboratories or commercial laboratories outside the country when ordering tests. This pathway is cumbersome and incurs high out-of-pocket costs for patients, underscoring the need for more feasibility data in this area. While only a limited number of genomics facilities in Nigeria offer sequencing services, the country’s genetics infrastructure and resources are primarily optimized for infectious diseases, like Lassa fever, and other prevalent conditions, such as sickle cell disease [26]. This is understandable considering the other urgent competing public health priorities in Nigeria and other SSA countries [27]. However, given that testing capabilities already exist in Nigeria, there is potential to capitalize on existing laboratory infrastructure to optimize the use of next-generation sequencing technologies for breast cancer. This approach could serve as a pathway to reducing global disparities in hereditary cancer management, by improving access to testing, enhancing the referral processes, and facilitating earlier detection and personalized treatment.

Lastly, our survey also identified a considerable gap in HCP professional training as another significant barrier to genetic testing in Nigeria. Less than 10% of respondents had received a formal training in genetics, which is linked to the distinct shortage of trained genetic counsellors. Providing tailored training opportunities in genetic counselling would equip HCPs with the necessary skills to quantify cancer risk, correctly document family histories, explain the risks and benefits of genetic testing, and offer psychosocial support. Effective genetic counselling has been shown to aid in interpretation of test results and guide decisions on follow-up care, including risk management and preventative options [28]. The scarcity of formal genetic counselling courses in Nigeria underscores the critical need for more trained professionals in this field, given the growing demand for hereditary cancer genetic services across the country [18]. Although most medical schools in Nigeria typically cover genetics within basic medical science courses, practical clinical genetics teaching is uncommon, and many skilled HCPs obtained their training abroad through fellowships or short courses [29]. Despite this, there is a high willingness to improve understanding in clinical genetics of breast cancer, with 71.9% of HCPs supporting the idea of a three-month intensive training period. Since there is no separate cadre of clinical geneticists or genetic counsellors in Nigeria, the most appropriate and cost-effective means for providing genetic counselling services is through training non-genetics clinicians [30]. Pilot data from Ibadan has shown that it is feasible to train nurses to provide genetic counselling [18], supporting a decentralized approach to address the existing counsellor shortage.

### Limitations

There are some inherent limitations to this study. The recruitment of participants for this survey study was conducted primarily through social media platforms; as a result, some potential participants may not have been reached. As with any survey of HCPs, a lower response rate is also a limiting factor. Finally, there may be bias in the responses as some Nigerian HCPs may have low genetic literacy, which might not allow for a sufficiently nuanced understanding of the genetics-based questions. Similarly, personal bias may impact on selection of perceived barriers.

## Conclusion

This study underscores the critical need for systemic improvements in breast cancer clinical genetics services within Nigeria from the perspective of healthcare providers. While our study shows relatively high awareness and accurate knowledge of NCCN-recommended criteria for hereditary breast cancer care, the integration into clinical practice is hindered by significant barriers. Most respondents reported a lack of testing infrastructure, referral pathways, genetic counselling services, and interprofessional training programs. Nonetheless, there is a willingness to undertake comprehensive training programs in genetic testing, counselling, and clinical management to improve access to genetic services across the country. These findings highlight critical barriers that must be addressed to integrate genetic testing into routine breast cancer care in Nigeria. Addressing the shortage of genetic counsellors may require exploring sustainable service delivery models, such as training non-genetics clinicians. This approach could bridge the gap between knowledge and practice of hereditary genetic services in Nigeria and align with global healthcare standards for personalized breast cancer treatment.

## Supplementary Information

Supplementary Material 1.

Supplementary Material 2.

Supplementary Material 3.

## Data Availability

No datasets were generated or analysed during the current study.

## Abbreviations

ASCO: American society of clinical oncology
BRCA 1: Breast cancer gene 1
BRCA 2: Breast cancer gene 2
HCP: Healthcare provider
LMIC: Low-and middle-income countries
NCCN: National comprehensive cancer network
RRSO: Risk-reducing salpingo-oophorectomy
SSA: Sub-Saharan Africa
