# Supplementary material for: Knowledge and perceptions of genetic testing for patients with breast cancer in Nigeria: a survey of healthcare providers
Source: Hered Cancer Clin Pract. 2025 May 19;23:16. doi: 10.1186/s13053-025-00315-w (PMC12087218; doi:10.1186/s13053-025-00315-w)
Supplement: Supplementary file 1 — Supplementary Material 1. [file 13053_2025_315_MOESM1_ESM.pdf]

Addressing global inequalities in breast cancer genetic counseling: Assessing the perception and knowledge of healthcare providers about genetic testing

Breast cancer is the most common malignancy in Nigerian patients, with an incidence of 54.3 per 100,000 population. The prevalence and penetrance of breast cancer susceptibility genes, including *BRCA1* & *BRCA2*, vary by population and remain poorly characterized outside of high-income populations. The *BRCA* status of the patient impacts treatment. This survey aims to assess the knowledge, perception, and barriers to breast cancer genetic testing in Nigeria.

Sociodemographics

1. Which group of health care providers do you belong to?
   Mark only one

   - ☐ Breast Surgical Oncologist
   - ☐ General Surgeon
   - ☐ Breast Radiologist
   - ☐ Clinical Oncologist
   - ☐ Radiation Oncologist
   - ☐ Nurse Oncologist
   - ☐ General Radiologist
   - ☐ Other: ________

2. Level of specialization:
   Mark only one

   - ☐ Senior Resident
   - ☐ Consultant
   - ☐ Oncology Nurse

3. Setting of practice:
   Mark only one

   - ☐ Private Hospital
   - ☐ Public Hospital (Teaching)
   - ☐ Public Hospital (Non-teaching)
   - ☐ Secondary Health care facility
   - ☐ Other: ________

4. The geopolitical zone of practice in Nigeria:
   Mark only one

   - ☐ North West
   - ☐ North East

- ☐ North Central
- ☐ South West
- ☐ South South
- ☐ South East

5. Age:
   Mark only one

   - ☐ <30 years
   - ☐ 31–44 years
   - ☐ 45–64 years
   - ☐ >65 years

6. Gender:
   Mark only one

   - ☐ Male
   - ☐ Female
   - ☐ Prefer not to say

7. How many breast cancer patients do you attend to in a month?
   Mark only one

   - ☐ 1–10 patients
   - ☐ 11–20 patients
   - ☐ >21 patients

8. In your practice, do you have any provision for genetic counseling?
   Mark only one

   - ☐ Yes
   - ☐ No
   - ☐ Maybe

9. In your practice, do you have any pathways for genetic counseling referral?
   Mark only one

   - ☐ Yes
   - ☐ No

Knowledge about Hereditary Breast Cancer

10. What percentage of breast cancer patients are likely to have a mutated *BRCA* gene?
    Mark only one

    - ☐ <5%
    - ☐ 5–10%
    - ☐ 10%

11. Amongst the patients listed below, who will require genetic testing?
    Check all that apply

    - ☐ Patient with a family history of breast cancer
    - ☐ Patient with multiple malignancies
    - ☐ Patient younger than 50 years
    - ☐ Patient with triple-negative breast cancer
    - ☐ All of the above
    - ☐ None of the above

12. Do you routinely discuss genetic testing with your patients?
    Mark only one

    - ☐ Yes
    - ☐ No

13. If no, why? ______________________

Perceptions about Genetic Testing for Hereditary Breast Cancer

14. Do you think *BRCA* testing will influence breast cancer care?
    Mark only one

    - ☐ Yes
    - ☐ No

15. If yes, how? ______________________

16. If no, how? ______________________

17. How do you perceive the usefulness of hereditary breast cancer diagnosis?
    Mark only one

	1	2	3	4	5	
Useful	☐	☐	☐	☐	☐	Not Useful

18. In the last 3 months, how often have you collected a complete family history from a breast cancer patient that includes the following: three generation disorders, age at diagnosis and death of each of the affected family member?
Mark only one

- ☐ Occasionally
- ☐ Sometimes
- ☐ Often
- ☐ Always
- ☐ Never

19. In the last 3 months, have any of your breast cancer patients initiated a discussion about genetic testing?
Mark only one

- ☐ Occasionally
- ☐ Sometimes
- ☐ Often
- ☐ Always
- ☐ Never

20. In the last 3 months, how often have you used family history information when facilitating clinical decisions or recommendations for your patients?
Mark only one

- ☐ Occasionally
- ☐ Sometimes
- ☐ Often
- ☐ Always
- ☐ Never

Barriers to Genetic Testing

21. Have you ever sent a patient for *BRCA* testing?
    Mark only one

    - ☐ Yes
    - ☐ No

22. If yes to above question, where would you send them? ______________________

23. How often do you send patient for *BRCA* testing?
    Mark only one

    - ☐ Never
    - ☐ Rarely
    - ☐ Often

24. If you selected never, would you be willing to send patients for BRCA testing?
    Mark only one

    - ☐ Yes
    - ☐ No
    - ☐ Maybe

25. Are you aware of any Laboratory conducting *BRCA* testing in Nigeria?
    Mark only one

    - ☐ Yes
    - ☐ No

26. If yes, where? ______________________

27. Do you have a genetic counsellor in your health care facility?
    Mark only one

    - ☐ Yes
    - ☐ No

28. What are the barriers that influence your choice of conducting *BRCA* testing for your patients?
    Check all that apply

    - ☐ Not aware of need for genetic testing
    - ☐ Lack of funding

- Lack of testing centers
- Patient not interested
- No reason
- No genetic counsellor
- Other (kindly explain) ___________

Training

29. Do you have any formal training in genetic counseling?
    Mark only one

    - Yes
    - No

30. If yes to above, where did you do your training? ______________________

31. If no to above, will you be willing to attend a training on genetic counseling?
    Mark only one

    - Yes
    - No

32. Would you be willing to attend or send a member of your team to a course on genetic counselling?
    Mark only one

    - Yes
    - No
    - Maybe

33. Which mode of training do you prefer?
    Mark only one

    - Online
    - Onsite/in-person
    - Hybrid (online/in-person)
    - Others (please explain)_____________

34. What length of training do you deem adequate to feel confident in referring patients for genetic counseling?
    Mark only one

- ☐ 3 months
- ☐ 3–6 months
- ☐ >6 months

---

35. Thank you for your time. Any other comments ____________
