# Supplementary material for: Knowledge and perceptions of genetic testing for patients with breast cancer in Nigeria: a survey of healthcare providers
Source: Hered Cancer Clin Pract. 2025 May 19;23:16. doi: 10.1186/s13053-025-00315-w (PMC12087218; doi:10.1186/s13053-025-00315-w)
Supplement: Supplementary file 2 — Supplementary Material 2. [file 13053_2025_315_MOESM2_ESM.pdf]

Supplementary Table 1: Demographic factors associated with knowledge of hereditary genetic testing for breast cancer

**Variables**	**Incorrect, n=50 (%)**	**Correct, n=71 (%)**	**p-value**
**Gender**			0.49
Male	30 (60.0)	47 (66.2)	
Female	19 (38.0)	24 (33.8)	
Prefer not to say	1 (2.0)	0 (0.0)	
**Age**			0.052
≤30years	3 (6.0)	1 (1.4)	
31-44years	27 (54.0)	52 (73.2)	
45-64years	20 (40.0)	17 (23.9)	
≥64years	0 (0.0)	1 (1.4)	
**Types of Practice Settings**			0.21
Private	5 (10.0)	3 (4.2)	
Public/Teaching	36 (72.0)	60 (84.5)	
Public/Non-Teaching Hospital	9 (18.0)	8 (11.3)	
**Health Care Provider Group**			0.094
Breast Surgical Oncologist	0 (0.0)	4 (5.6)	
General Surgeon	22 (44.0)	32 (45.1)	
Clinical and Radiation Oncologist	9 (18.0)	20 (28.2)	
Breast Radiologist	1 (2.0)	2 (2.8)	
Nurse Oncologist	18 (36.0)	13 (18.3)	
**Geopolitical Zone**			0.076
North Central	17 (34.0)	13 (18.3)	
North East	4 (8.0)	2 (2.8)	
North West	7 (14.0)	14 (19.7)	
South South	4 (8.0)	4 (5.6)	
South West	13 (26.0)	34 (47.9)	
South East	5 (10.0)	4 (5.6)	
**Number of patients with breast cancer patients seen per month**			0.28
1-10 patients	25 (50.0)	27 (38.0)	
11-20 patients	16 (32.0)	23 (32.4)	
≥ 21patients	9 (18.0)	21 (29.6)	
