# Supplementary material for: Knowledge and perceptions of genetic testing for patients with breast cancer in Nigeria: a survey of healthcare providers
Source: Hered Cancer Clin Pract. 2025 May 19;23:16. doi: 10.1186/s13053-025-00315-w (PMC12087218; doi:10.1186/s13053-025-00315-w)
Supplement: Supplementary file 3 — Supplementary Material 3. [file 13053_2025_315_MOESM3_ESM.pdf]

Supplementary Table 2: Demographic factors associated with perceived usefulness of a hereditary breast cancer diagnosis

Variables	Perception Rating Mean (sd)	Estimate (95%CI)	p-value
**Gender**			
Male	1.9 (1.3)	Reference	
Female	1.6 (1.1)	-0.3 (-0.7, 0.2)	0.28
**Age**			
≤30years	1.8 (1.5)	Reference	
31-44years	1.7 (1.2)	-0.003 (-1.2, 1.2)	1.00
45-64years	1.9 (1.3)	0.2 (-1.1, 1.5)	0.76
**Types of Practice Settings**			
Private	1.6 (1.1)	Reference	
Public/Teaching	1.8 (1.2)	0.2 (-0.7, 1.2)	0.65
Public/Non-Teaching Hospital	2.0 (1.1)	0.4 (-0.7, 1.5)	0.44
**Health Care Provider Group**			
Breast Surgical Oncologist	1.2 (0.5)	Reference	
General Surgeon	2.2 (1.4)	1.0 (-0.2, 2.2)	0.12
Clinical and Radiation Oncologist	1.4 (0.8)	0.1 (-1.1, 1.4)	0.84
Breast Radiologist	1.0 (0.0)	-0.3 (-2.0, 1.5)	0.78
Nurse Oncologist	1.7 (1.0)	0.4 (-0.8, 1.6)	0.51
**Geopolitical Zone**			
North Central	1.9 (1.2)	Reference	
North East	1.0 (0.0)	-0.9 (-2.0, 0.1)	0.081
North West	1.7 (1.1)	-0.3 (-0.9, 0.4)	0.43
South South	2.6 (1.7)	0.7 (-0.2, 1.6)	0.14
South West	1.6 (1.1)	-0.3 (-0.9, 0.2)	0.27
South East	2.4 (1.6)	0.5 (-0.4, 1.4)	0.26
**Number of patients with breast cancer patients seen per month**			
1-10 patients	1.9 (1.3)	Reference	
11-20 patients	1.9 (1.3)	0.0 (-0.5, 0.5)	0.95
≥ 21patients	1.4 (0.9)	-0.5 (-1.1, 0.0)	0.062
